# Sickle cell disease in Germany – trends in epidemiology, healthcare utilization and disease burden from 2015 to 2024: a claims data analysis

**DOI:** 10.1007/s00277-026-07308-2

**Published:** 2026-10-23

**Authors:** Nils Schulze, Moritz Lehne, Anja Cengia, Klara Sonnie Hinrichsmeyer, Agnes Kisser, Ferras Alashkar, Caoimhe Cawley

**Affiliations:** 1 Institute of Health Services and Health Systems Research, Immanuel Clinic Rüdersdorf, Medical University Brandenburg Theodor Fontane, Seebad 82/83, 15562 Rüdersdorf bei Berlin, Germany; 2 Pfizer Pharma GmbH, Friedrichstraße 110, 10117 Berlin, Germany; 3 Cytel Inc, Potsdamer Straße 58, 10785 Berlin, Germany; 4 InGef – Institute for Applied Health Research Berlin GmbH, Otto-Ostrowski- Straße 5, 10249 Berlin, Germany; 5 Department of Hematology and Stem Cell Transplantation, West German Cancer Center, University of Duisburg-Essen, University Hospital Essen, Hufelandstraße 55, 45147 Essen, Germany

**Keywords:** Sickle cell disease, Claims data, Epidemiology, Prevalence, Incidence, Healthcare resource utilization (HCRU)

## Abstract

Background: Sickle cell disease (SCD) is a rare inherited hemoglobinopathy associated with substantial morbidity and high healthcare needs. In Germany, population-based evidence on SCD epidemiology and real-world care patterns remains limited, despite increasing relevance in the context of migration and the introduction of nationwide newborn screening in 2021. Methods: We conducted a retrospective, non-interventional study using anonymized German statutory health insurance claims data from the InGef database (2015–2024). Patients with SCD were identified using ICD-10-GM codes D57.0, D57.1, D57.2, and D57.8 based on inpatient and confirmed outpatient diagnoses. Administrative prevalence was estimated using a carry-forward cohort approach, whereby SCD patients diagnosed in the current year and alive/observable in subsequent years were counted as prevalent. Administrative incidence was assessed in children aged < 6 years using the first recorded SCD diagnosis in claims data as the date of incidence. Analyses of patient characteristics, clinical outcomes, treatments and healthcare resource use (HCRU) were conducted cross-sectionally by calendar year, stratified by age group and sex. Results: Across 2015–2024, 1,019 individuals with SCD were identified. As might be expected using a carry forward cohort approach, annual prevalent counts increased from 225 (2015) to 864 (2024), and nationally extrapolated prevalence increased from 2.75 per 100,000 in 2015 to 10.50 per 100,000 in 2024. Median time to recorded diagnosis in children < 6 years decreased from 31 months (2015) to 11 months from 2021 onwards (2021–2024). In 2024, infections were the most frequently documented clinical outcome (34.5% [298/864]), followed by vaso‑occlusive crises (19.2% [166/864]) and mental health comorbidities (depression 19.1% [165/864]; anxiety 9.1% [79/864]). The proportions of patients with inpatient or outpatient visits, and with medication prescriptions (e.g. for antibiotics, antirheumatics, hydroxyurea), decreased over time. Conclusion: This claims-based analysis indicates increasing documented SCD prevalence in Germany. However, the extent to which prevalence increased due to a true increase in the general population, due to our carry-forward cohort approach, or due to other factors such as changes in coding practices or healthcare seeking behaviors, cannot be disentangled.We observed earlier recorded diagnosis timing in young children over time, alongside substantial age-dependent burden reflected in complications, mental health comorbidity, and healthcare utilization. Possible explanations for lower proportions of patients with inpatient/outpatient visits and medication prescriptions over time may include changes in disease awareness, patient management and/or healthcare seeking behaviours over time, and/or broader capture of patients with milder disease. Overall, our findings can inform healthcare planning and structured life-course–oriented SCD care pathways in Germany.

**Supplementary Information:**

The online version contains supplementary material available at https://doi.org/10.1007/s00277-026-07308-2.

## Introduction

Sickle cell disease (SCD) is an inherited hemoglobinopathy caused by a point mutation in the β‑globin gene, leading to hemoglobin S polymerization, erythrocyte sickling, vaso‑occlusion, hemolysis, and progressive multi‑organ damage [1]. Clinically, SCD is characterized by acute complications such as vaso‑occlusive crises (VOCs), acute chest syndrome, and stroke, as well as chronic sequelae affecting multiple organ systems [2, 3]. Despite advances in supportive and preventive care, SCD remains associated with substantial morbidity, psychosocial burden, and reduced survival [1, 3].

In Germany, SCD has gained increasing relevance in recent decades in the context of migration and changing population composition. Available estimates vary by data source and case ascertainment. Based on a national registry publication, the minimum number of individuals living with SCD in Germany was estimated to be at least 2,000 in 2017, while acknowledging that the true number may be higher due to under‑ascertainment, particularly among adults and milder cases [4]. A population‑based statutory health insurance claims analysis estimated 2,005 individuals with SCD in 2011 and 3,160 in 2019 [5]. Claims‑based newborn analyses further suggest that approximately 70–150 children with SCD may be born each year in Germany [6]. Together, these findings underline both the increasing policy relevance of SCD and the continued uncertainty regarding national epidemiologic burden.

To improve early detection and timely initiation of preventive measures, SCD was incorporated into Germany’s nationwide expanded newborn screening program in 2021 [7]. However, clinical and health‑system challenges extend beyond early diagnosis. Long‑term disease management requires coordinated multidisciplinary care, prevention and treatment of complications, and continuity across life stages.

Despite this growing clinical and policy relevance, population‑based evidence describing SCD epidemiology and disease burden in Germany remains limited and fragmented across data sources. Routinely collected statutory health insurance claims data provide an opportunity to assess recorded (“administrative”) SCD epidemiology and routine care patterns at scale. We therefore conducted a retrospective descriptive analysis using the InGef claims database covering 2015–2024. Our objectives were to describe temporal trends in administrative prevalence and administrative incidence (in children aged < 6 years), including time to recorded diagnosis, and to characterize demographics, clinical outcomes, treatments, and healthcare resource utilization (HCRU) over time and by age group.

## Methods

### Study design and data source

We conducted a retrospective, non‑interventional observational study using anonymized statutory health insurance claims data from the InGef database (Institut für angewandte Gesundheitsforschung Berlin), covering the period from 01/01/2015 to 12/31/2024. The InGef database comprises routinely collected reimbursement data and includes inpatient and outpatient diagnoses (ICD‑10‑GM), outpatient prescriptions (ATC/PZN), procedures (OPS), insurance periods, length of stay, and mortality information. The database includes approximately eight million individuals per year. Comparisons of the InGef database with national population data show a close correlation in terms of gender distribution, but with middle-aged adults and insured persons from the western federal states being slightly over-represented, and older age-groups slightly under-represented [8]. Within the database, which includes records from more than 50 different health insurance funds, individuals are assigned fixed pseudonyms, which enable longitudinal linking across calendar years and also between the different health insurance funds that submit data to the InGef database. Data records following a break in insurance cover can be linked to earlier observation periods using the same pseudonym. Reporting followed STROBE and the RECORD extension for studies using routinely collected health data [9, 10].

### Study population and SCD case definition

Patients with sickle cell disease (SCD) were identified using ICD‑10‑GM codes D57.0, D57.1, D57.2, and D57.8 based on inpatient and confirmed outpatient diagnoses. The sickle cell trait code D57.3 was not used to define SCD cases. Individuals were considered SCD cases if they met at least one of the following criteria during 2015–2024: (i) ≥ 1 primary inpatient diagnosis of SCD; (ii) ≥ 1 confirmed outpatient diagnosis of SCD documented by a hematology specialty code (German “Fachgruppencode” 27, 35, or 41); or (iii) ≥ 2 confirmed outpatient diagnoses documented by two independent physicians, irrespective of specialty. For inclusion in the annual prevalence cohorts, at least one of the case criteria had to be met in the current calendar year. The fulfillment of a case criterion later did not result in retrospective inclusion in earlier annual cohorts. For criterion (iii), a case was only classified in the calendar year in which the second confirmed outpatient SCD diagnosis had been documented. In order to record outpatient cases, only SCD diagnoses marked with the German diagnosis certainty modifier ‘G’ (‘confirmed’) were extracted. Diagnoses marked with ‘V’ (‘suspected’), ‘A’ (‘excluded’) or ‘Z’ (‘post’) were not extracted.

### Epidemiological measures

#### Administrative prevalence

Annual administrative prevalence was estimated for each calendar year (2015–2024) using a carry‑forward cohort approach appropriate for a lifelong inherited condition: patients with an SCD diagnosis in the current year or any subsequent years in which the patient was alive/observable during any quarter of that year were counted as prevalent. The denominator comprised all individuals in the database who were observable and alive during any quarter of the respective calendar year. Observability was determined on the basis of the documented periods of insurance and did not require the existence of individual claims for services.

#### Administrative incidence (< 6 years)

Administrative incidence was assessed among children aged < 6 years using the first recorded SCD diagnosis during the study period as the incident date, in order to approximate true incidence, which happens at birth. Because we did not use a look back period, for the year 2015 incident and prevalent cases could not be distinguished. For this reason, administrative incidence estimates are presented starting from the year 2016.

#### Time to recorded diagnosis (< 6 years)

Among children aged < 6 years, time to recorded diagnosis was assessed as the time in months from the start of observability to the first recorded SCD diagnosis in claims data. Results were summarized annually using the median; additional distribution measures are provided in Online Resource 2. Supplementary analyses for a more restrictive full-coverage cohort (continuous observability from birth) are provided in Online Resource 2 for transparency and sensitivity assessment.

#### National extrapolation and hospital‑correction sensitivity analysis

Administrative prevalence and incidence were reported per 100,000 and extrapolated to the German national population using age‑ and sex‑specific statistics from the German Federal Statistical Office (Destatis). As a sensitivity analysis addressing potential over‑ or under‑representation of SCD in the InGef sample, we additionally applied a hospital‑diagnosis–based correction factor using Destatis hospital discharge statistics: annual national numbers of hospital admissions with a principal diagnosis of SCD (ICD‑10‑GM D57.0/D57.1/D57.2/D57.8) were extracted and compared to the corresponding national number of SCD‑related admissions estimated based on study sample, using a matching hospitalization definition. The ratio of Destatis admissions to study‑estimated admissions was used as a correction factor and applied to the national extrapolated administrative prevalence and administrative incidence estimates.

### Clinical outcomes and healthcare resource utilization (HCRU)

Clinical outcomes and HCRU, including treatment utilization, were assessed cross-sectionally for each individual study year (2015–2024) and summarized as the number and proportion of patients with ≥ 1 documented event or exposure in the respective year. Definitions and complete ICD/OPS/ATC code lists for all outcomes, HCRU variables, and treatment categories are provided in Online Resource 1.

#### Clinical outcomes

Key clinical symptoms and complications were identified based on diagnoses and/or procedure codes, as applicable. Because VOCs are not directly coded in German claims data, they were estimated using a predefined proxy algorithm based on hospital admissions, which is based on an earlier analysis of data from the statutory health insurance system in Germany [11]. Briefly, VOCs were identified from SCD-related inpatient hospitalizations reflecting acute vaso-occlusive or pain-related events; acute chest syndrome and priapism were considered VOC-related acute complications within this operational definition. Full operational details and code lists are provided in Online Resource 1.

#### HCRU

HCRU comprised outpatient contacts, hospitalizations, inpatient length of stay, and treatment utilization. Outpatient contacts were differentiated as all-cause and SCD-related based on diagnoses and physician specialty codes. Hospitalizations were classified as all-cause or SCD-related based on diagnoses. Inpatient length of stay (LoS) was summarized as mean (SD) for all-cause and SCD-related stays. Treatment utilization was identified using outpatient prescription data (ATC/PZN) and relevant procedure codes (OPS/EBM), where applicable, and summarized within treatment categories.

### Stratification, summary measures, and small‑cell suppression

Administrative prevalence estimates and all burden measures (clinical outcomes and HCRU, including treatment utilization) were stratified by sex and age group (0–17 years and ≥ 18 years). Age was determined as of 1 January of the respective calendar year. All analyses were descriptive; no formal hypothesis testing was conducted. To comply with data protection requirements, small cell counts were suppressed as < 5 and corresponding percentages were not displayed.

### Ethics and data protection

Analyses were based on anonymized secondary claims data and conducted in accordance with German legal and regulatory requirements, including § 75 SGB X. An ethics waiver was documented (approval number: 305052025ANF).

## Results

### Study population

Across 2015–2024, 1,019 individuals with sickle cell disease (SCD) were identified in the InGef database. Overall, 57.1% (582/1,019) were female and 72.6% (740/1,019) were aged ≥ 18 years; the median age was 33 years (IQR 16–48). The annual number of prevalent SCD patients increased from 225 in 2015 to 864 in 2024 (Table 1). Baseline characteristics by calendar year are summarized in Table 2, including age groups, sex, insurance status, and recorded ICD‑10‑GM subcodes for SCD.

**Table 1:** Sample selection, administrative prevalence (all age groups), and administrative incidence (children aged < 6 years) in the InGef database, 2015 to 2024

Year	Total sample	Prevalent SCD patients*, *N*	Nationally extrapolated prevalence per 100,000, (95% CI)**	Adjusted nationally extrapolated prevalence per 100,000; (95% CI)***	SCD patients aged < 6 years, *N*	Nationally extrapolated administrative incidence (per 100,000); (95% CI)**	Adjusted nationally extrapolated incidence (per 100,000); (95% CI)***
2016	7,751,502	332	4.02 (3.88–4.15)	3.85 (3.72–3.98)	8	1.52 (1.20–1.93)	1.58 (1.13–2.22)
2017	7,760,633	422	5.07 (4.92–5.23)	4.82 (4.68–4.98)	11	2.07 (1.70–2.54)	2.10 (1.55–2.85)
2018	7,783,713	486	5.93 (5.77–6.10)	5.69 (5.53–5.85)	11	2.19 (1.80–2.65)	2.16 (1.66–2.82)
2019	7,816,098	543	6.64 (6.47–6.82)	6.48 (6.31–6.66)	8	1.56 (1.25–1.96)	1.51 (1.14–2.01)
2020	7,790,977	606	7.44 (7.26–7.63)	7.29 (7.11–7.48)	6	1.20 (0.93–1.55)	1.15 (0.81–1.64)
2021	7,840,994	674	8.26 (8.07–8.46)	8.10 (7.91–8.30)	15	1.52 (1.21–1.92)	3.52 (2.77–4.49)
2022	7,891,448	751	9.17 (8.96–9.37)	9.09 (8.89–9.30)	15	2.80 (2.36–3.33)	2.78 (2.29–3.38)
2023	7,953,871	809	9.86 (9.65–10.07)	9.74 (9.53–9.96)	12	3.00 (2.54–3.55)	3.20 (2.45–4.17)
2024	7,991,879	864	10.50 (10.28–10.72)	10.40 (10.18–10.62)	12	2.29 (1.89–2.78)	2.27 (1.84–2.81)

Notes:

* At least one primary inpatient diagnosis and/or ≥ 1 confirmed outpatient diagnosis of sickle cell disease (ICD-10-GM: D57.0, D57.1, D57.2, D57.8) by a hematologist (German specialty code [“Fachgruppencode”]: 27, 35, 41) or ≥ 2 confirmed outpatient diagnoses by two independent physicians in the study period between 01/01/2015 and 31/12/2024

**Extrapolated using age- and sex-specific statistics from the German Federal Statistical Office

***Extrapolated using age- and sex-specific statistics from the German Federal Statistical Office, as well as the ratio of the number of SCD hospitalizations within the InGef sample compared to the national number of SCD hospitalizations (“hospital correction factor”)

**Table 2:** Baseline characteristics

Characteristic			2015	2016	2017	2018	2019	2020	2021	2022	2023	2024
N (%)		All patients	225 (100)	332 (100)	422 (100)	486 (100)	543 (100)	606 (100)	674 (100)	751 (100)	809 (100)	864 (100)
		0–17 years	57 (25.3)	81 (24.4)	104 (24.6)	120 (24.7)	125 (23.0)	124 (20.5)	137 (20.3)	149 (19.8)	166 (20.5)	181 (20.9)
		≥ 18 years	168 (74.7)	251 (75.6)	318 (75.4)	366 (75.3)	418 (77.0)	482 (79.5)	537 (79.7)	602 (80.2)	643 (79.5)	683 (79.1)
Female, n (%)		All patients	134 (59.6)	191 (57.5)	238 (56.4)	271 (55.8)	305 (56.2)	342 (56.4)	389 (57.7)	433 (57.7)	464 (57.4)	493 (57.1)
		0–17 years	31 (54.4)	44 (54.3)	52 (50.0)	57 (47.5)	57 (45.6)	50 (40.3)	59 (43.1)	69 (46.3)	81 (48.8)	86 (47.5)
		≥ 18 years	103 (61.3)	147 (58.6)	186 (58.5)	214 (58.5)	248 (59.3)	292 (60.6)	330 (61.5)	364 (60.5)	383 (59.6)	407 (59.6)
Median age (years) (IQR)		All patients	36 (17–49)	38 (19–50)	37 (18–50)	37 (18–51)	37 (18–51)	38 (20–52)	40 (21–53)	39 (21–54)	40 (21–54)	40 (21–55)
		0–17 years	9 (4–13)	10 (5–14)	10 (4–14)	9 (4–14)	8 (4–13)	9 (5–13)	9 (5–13)	9 (5–13)	9 (4–13)	9 (4–13)
		≥ 18 years	44 (32–51)	45 (33–52)	44 (33–52)	45 (32–54)	45 (32–54)	45 (32–55)	46 (32–56)	46 (32–56)	46 (33–57)	46 (33–58)
Health insurance status, n (%)	insured	All patients	116 (51.6)	179 (53.9)	235 (55.7)	270 (55.6)	312 (57.5)	365 (60.2)	400 (59.4)	443 (59.0)	480 (59.3)	518 (60.0)
		0–17 years	*	5 (6.2)	6 (5.8)	6 (5.0)	7 (5.6)	9 (7.3)	7 (5.1)	6 (4.0)	7 (4.2)	10 (5.5)
		≥ 18 years	114 (67.9)	174 (69.3)	229 (72.0)	264 (72.1)	305 (73.0)	356 (73.9)	393 (73.2)	437 (72.6)	473 (73.6)	509 (74.5)
	family-insured	All patients	79 (35.1)	110 (33.1)	139 (32.9)	158 (32.5)	165 (30.4)	169 (27.9)	188 (27.9)	208 (27.7)	220 (27.2)	228 (26.4)
		0–17 years	53 (93.0)	73 (90.1)	96 (92.3)	112 (93.3)	115 (92.0)	111 (89.5)	126 (92.0)	138 (92.6)	154 (92.8)	161 (89.0)
		≥ 18 years	26 (15.5)	37 (14.7)	43 (13.5)	46 (12.6)	50 (12.0)	58 (12.0)	62 (11.6)	70 (11.6)	66 (10.3)	67 (9.8)
	retired	All patients	30 (13.3)	43 (12.9)	48 (11.4)	58 (11.9)	64 (11.8)	69 (11.4)	81 (12.0)	95 (12.7)	106 (13.1)	111 (12.9)
		0–17 years	*	*	*	*	*	*	*	*	5 (3.0)	5 (2.8)
		≥ 18 years	28 (16.7)	40 (15.9)	46 (14.5)	56 (15.3)	62 (14.8)	66 (13.7)	79 (14.7)	91 (15.1)	101 (15.7)	106 (15.5)
D57.0 (%)		All patients	35.1	28.3	27.7	22.6	19.7	18.6	18.1	17.4	16.4	16.7
		0–17 years	52.6	44.4	46.2	38.3	38.4	34.7	35.8	32.9	30.1	31.5
		≥ 18 years	29.2	23.1	21.7	17.5	14.1	14.5	13.6	13.6	12.9	12.7
D57.1 (%)		All patients	79.1	66.3	64.2	58.6	55.1	51.7	48.4	50.1	49.6	47.9
		0–17 years	89.5	72.8	70.2	68.3	57.6	56.5	48.9	54.4	58.4	50.3
		≥ 18 years	75.6	64.1	62.3	55.5	54.3	50.4	48.2	49.0	47.3	47.3
D57.2 (%)		All patients	19.1	18.1	14.7	14.4	13.6	12.5	13.5	12.0	11.1	9.4
		0–17 years	15.8	14.8	15.4	14.2	14.4	13.7	10.9	8.1	8.4	6.1
		≥ 18 years	20.2	19.1	14.5	14.5	13.4	12.2	14.2	13.0	11.8	10.2
D57.8 (%)		All patients	5.3	6.3	8.5	7.6	9.0	6.6	7.4	7.1	8.2	7.3
		0–17 years	*	11.1	10.6	10.8	12.8	9.7	15.3	14.8	16.3	14.9
		≥ 18 years	4.8	4.8	7.9	6.6	7.9	5.8	5.4	5.1	6.1	5.3

Note: For categorical variables, data are presented as n (%) within each age group and calendar year. Median age is reported as median (IQR). ICD‑10‑GM code categories are not mutually exclusive (multiple codes may be recorded per patient). Small cell counts are suppressed as < 5; suppressed values are indicated by “*”

### Administrative prevalence (2015–2024)

**Fig. 1:**
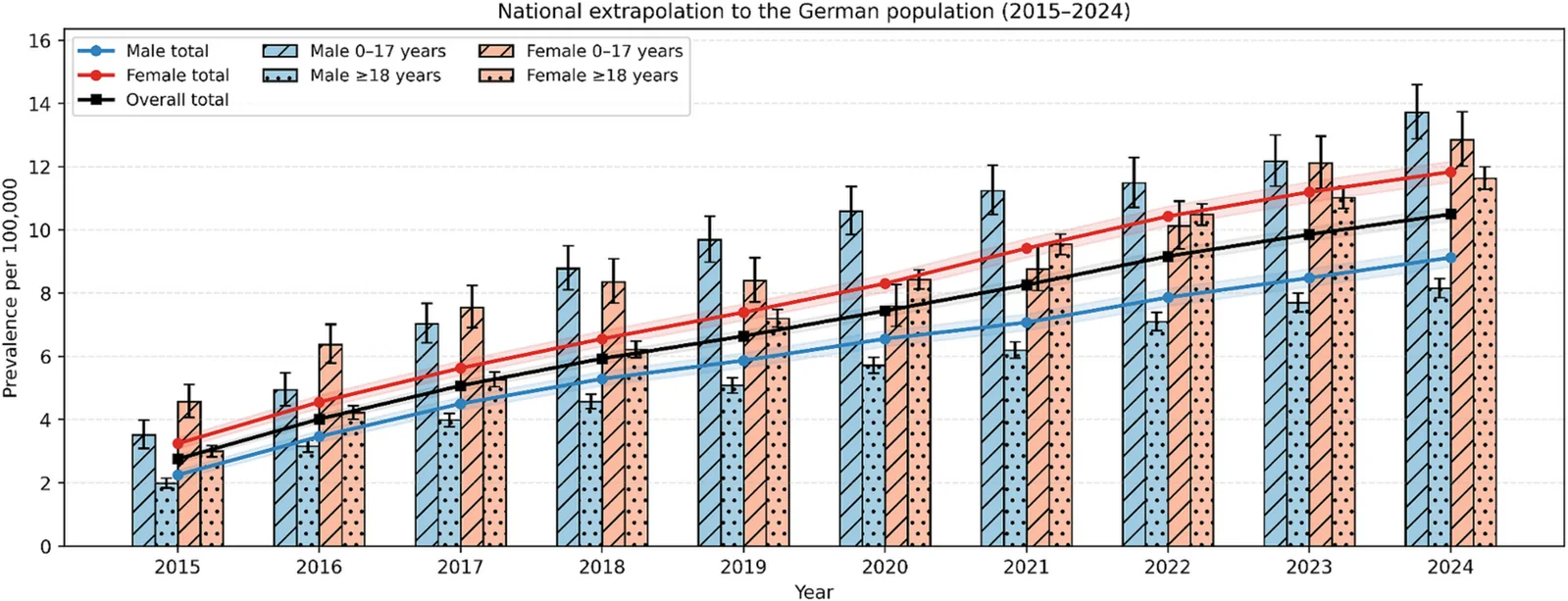
Nationally extrapolated administrative SCD prevalence per 100,000 population in Germany, overall and by age groups and sex 2015–2024

Nationally extrapolated administrative prevalence increased from 2.75 per 100,000 (95% CI 2.64–2.87) in 2015 to 10.50 per 100,000 (95% CI 10.28–10.72) in 2024 (Table 1; Fig. 1); hospital‑corrected (adjusted) estimates were very similar (Table 1; Fig. 1). Prevalence increased over time across all age and sex strata shown in Fig. 1, with higher prevalence in children/adolescents than in adults.

### Administrative incidence and time to recorded diagnosis in children aged < 6 years (2015–2024)

**Fig. 2:**
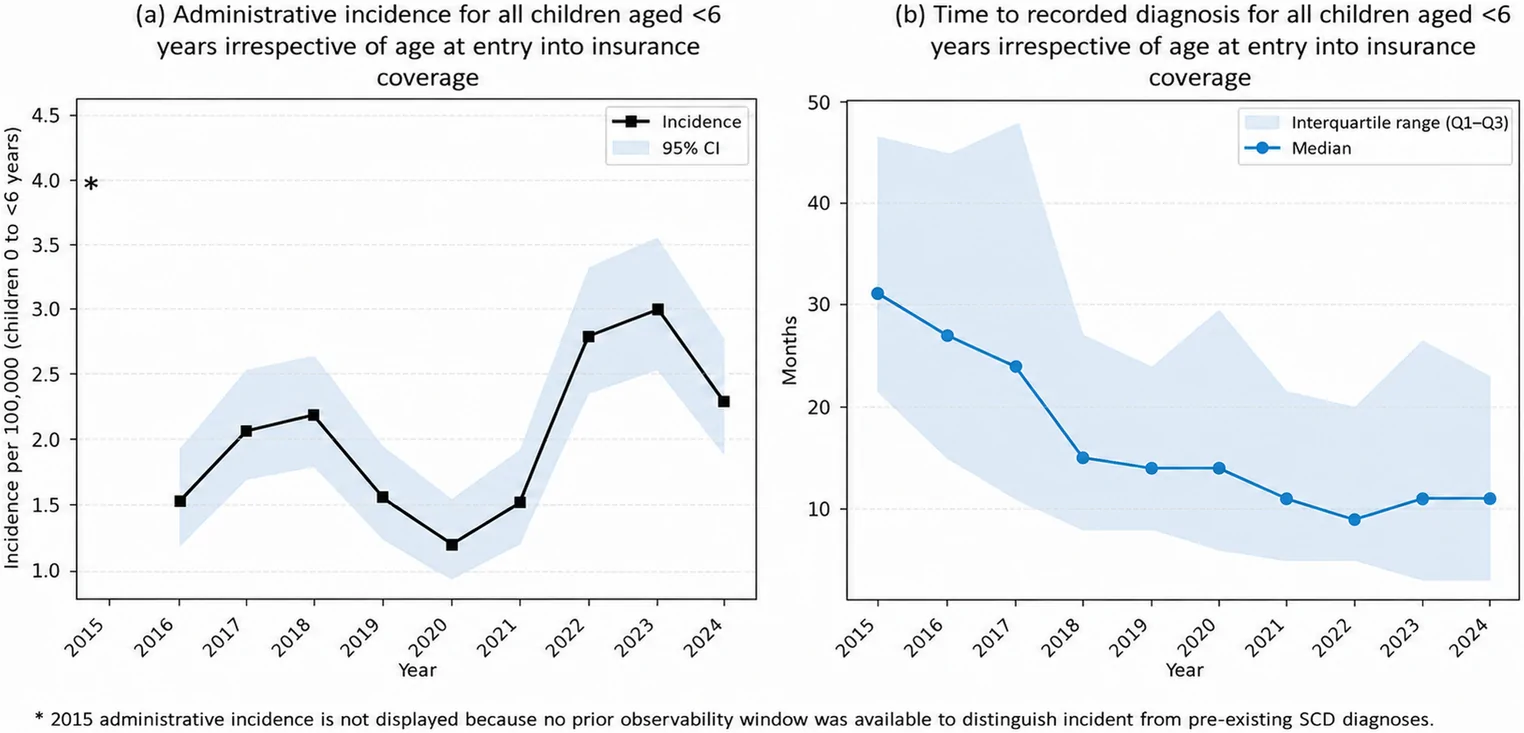
Temporal trends in children aged < 6 years, irrespective of age at entry into insurance coverage: (**a**) administrative incidence of SCD per 100,000 population and (**b**) median time to recorded diagnosis in months, 2015–2024

Administrative incidence estimates in children aged < 6 years are shown in Table 1; Fig. 2a. From 2016 onwards, annual administrative incidence remained in a comparable range, with some year-to-year variation; it was 1.52 per 100,000 (95% CI 1.20–1.93) in 2016, 1.20 per 100,000 (95% CI 0.93–1.55) in 2020, and 2.29 per 100,000 (95% CI 1.89–2.78) in 2024 (Table 1; Fig. 2a). A sharp rise is seen from 2021 (1.52 per 100,000, 95% CI 1.21–1.92) to 2022 (2.80 per 100,000, 95% CI 2.36–3.33) (Fig. 2a). However, it should be borne in mind that the estimates are based on small numbers of identified incident cases (9 in 2021, 15 in 2022). Hospital-corrected incidence estimates were similar in most years.

Median time to recorded diagnosis in children aged < 6 years decreased from 31 months in 2015 to < 12 months from 2021 onward (2021–2024) (Fig. 2b).

### Clinical outcomes (2015–2024)

**Fig. 3:**
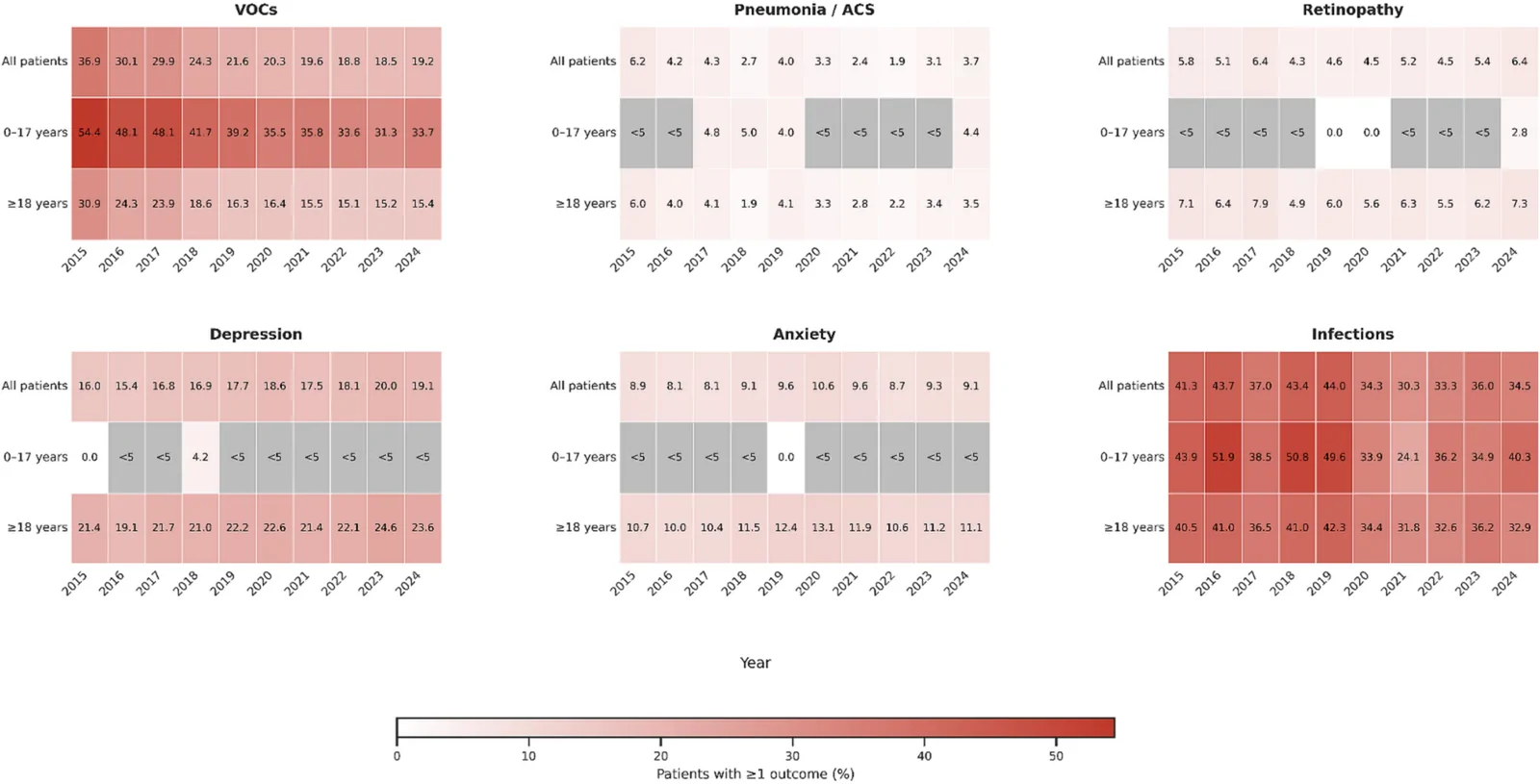
Clinical outcomes in patients with SCD (proportion of patients with ≥ 1 documented outcome) in Germany, 2015–2024, by age group. Grey cells indicate cell count < 5; white cells indicate zero observations

Infections were the most frequently documented outcome throughout the observation period, followed by VOCs, depression, and anxiety (Fig. 3; Online Resource 3). In all patients, infections decreased from 41.3% in 2015 to 34.5% in 2024 and were more frequently documented in children/adolescents than in adults in 2024 (40.3% vs. 32.9%).

Vaso-occlusive crises (VOCs; proxy definition) showed a decreasing pattern over time in all patients, from 36.9% in 2015 to 19.2% in 2024 (Fig. 3). VOCs were consistently more frequently documented in children/adolescents than in adults, including 54.4% vs. 31.0% in 2015 and 33.7% vs. 15.4% in 2024.

Mental health comorbidity was documented throughout the study period and was more frequently recorded in adults than in children/adolescents (Fig. 3). Depression in all patients was 16.0% in 2015 and 19.1% in 2024; anxiety was 8.9% in 2015 and 9.1% in 2024.

In adults, retinopathy remained relatively stable over time and was rare in children/adolescents. Pneumonia/acute chest syndrome remained comparatively uncommon overall, with several pediatric year-specific cells affected by small-cell suppression (Fig. 3).

### Healthcare resource utilization (HCRU) (2015–2024)

**Fig. 4:**
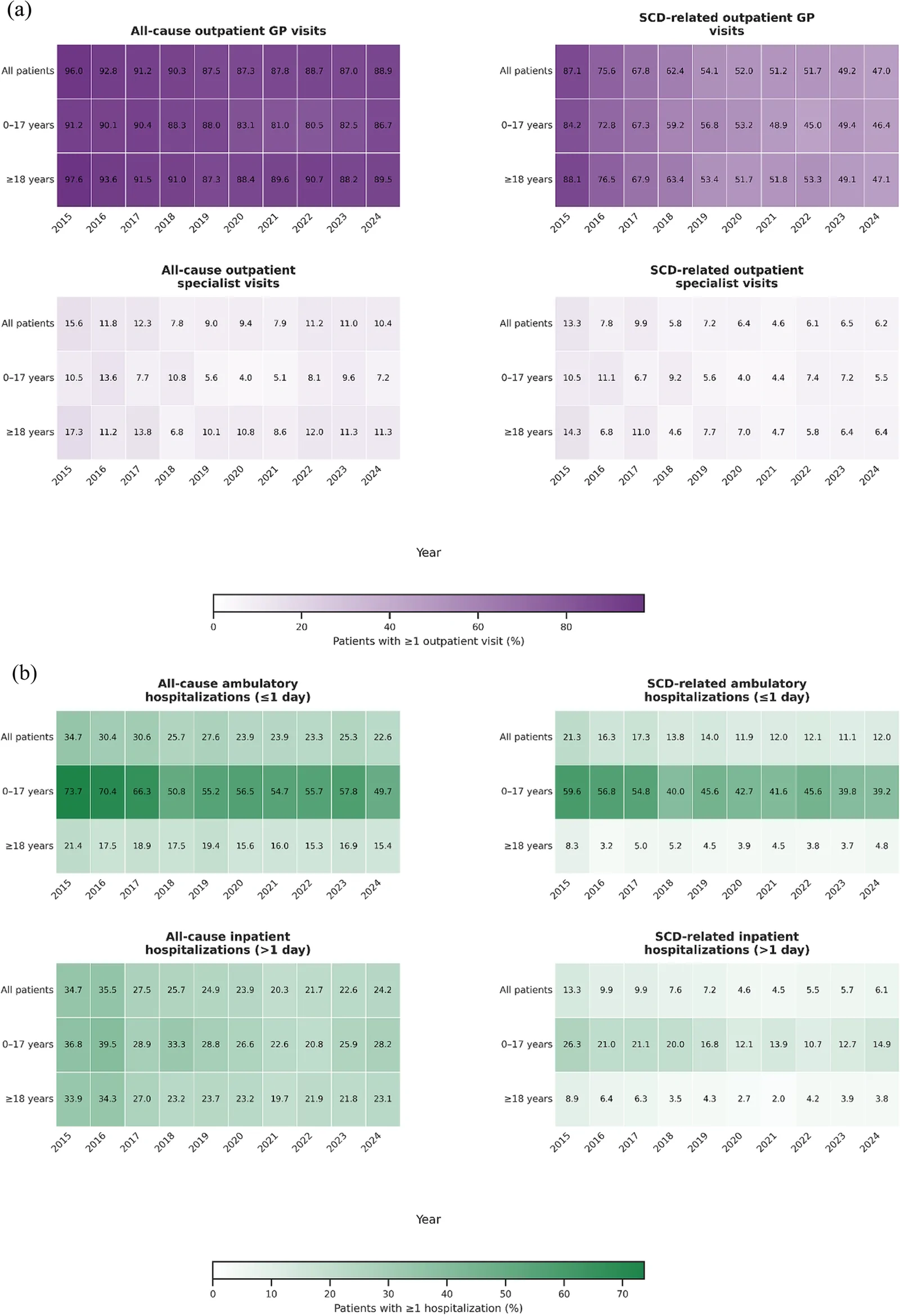
(**a**) Annual healthcare resource utilization (HCRU) in patients with SCD (proportion of patients with ≥ 1 outpatient contacts in the respective year) in Germany, 2015–2024, by age group and cause; (**b**) annual healthcare resource utilization (HCRU) in patients with SCD (proportion of patients with ≥ 1 hospitalization in the respective year) in Germany, 2015–2024, by age group

Outpatient GP contact remained common throughout the observation period, while the annual proportion of SCD-related GP contacts declined over time in both age groups (Fig. 4a).

Outpatient specialist visits were less frequent than GP visits. In all patients, all-cause outpatient specialist visits decreased from 15.6% in 2015 to 10.4% in 2024, and SCD-related outpatient specialist visits from 13.3% to 6.3% (Fig. 4a). In 2024, SCD-related outpatient specialist visits were similar in children/adolescents and adults (5.5% vs. 6.4%).

Hospital‑based HCRU showed clear age‑dependent patterns (Fig. 4b). All‑cause ambulatory hospitalizations (≤ 1 day) in all patients declined from 34.67% (78/225) in 2015 to 22.57% (195/864) in 2024; children/adolescents consistently had higher proportions than adults (e.g., 73.68% [42/57] vs. 21.43% [36/168] in 2015; 49.72% [90/181] vs. 15.37% [105/683] in 2024). SCD‑related ambulatory hospitalizations (≤ 1 day) declined from 21.33% (48/225) in 2015 to 12.04% (104/864) in 2024 and were markedly higher in children/adolescents than in adults (e.g., 59.65% [34/57] vs. 8.33% [14/168] in 2015; 39.23% [71/181] vs. 4.83% [33/683] in 2024).

A similar age gradient was observed for SCD‑related inpatient hospitalizations (> 1 day). In all patients, SCD‑related inpatient hospitalizations decreased from 13.33% (30/225) in 2015 to 6.13% (53/864) in 2024. Children/adolescents had consistently higher proportions than adults (e.g., 26.32% [15/57] vs. 8.93% [15/168] in 2015; 14.92% [27/181] vs. 3.81% [26/683] in 2024). 

In all patients, mean all-cause LoS was 6.82 days (SD 21.30) in 2015 and 5.54 days (SD 21.89) in 2024. Mean SCD-related LoS was 3.03 days (SD 13.78) in 2015 and 1.47 days (SD 9.82) in 2024 (Table 3). Age differences were most pronounced for hospital-based HCRU, with consistently higher SCD-related ambulatory and inpatient hospital use in children/adolescents than in adults. Further HCRU results are provided in Online Resource 3.

**Table 3:** Inpatient length of stay (all-cause and SCD-related) by age group

	*N* (annual prevalent cohort)			Length of stay (all-cause) (days); mean (SD)			Length of stay (SCD-related) (days); mean (SD)		
	All patients	0–17 years	≥ 18 years	All patients	0–17 years	≥ 18 years	All patients	0–17 years	≥ 18 years
Year									
2015	225	57	168	6.82 (21.30)	7.82 (19.29)	6.48 (21.99)	3.03 (13.78)	3.68 (9.21)	2.81 (15.04)
2016	332	81	251	8.50 (26.23)	6.11 (14.28)	9.27 (29.04)	1.34 (5.37)	2.36 (6.58)	1.01 (4.89)
2017	422	104	318	8.27 (33.90)	9.85 (43.39)	7.75 (30.23)	2.50 (18.24)	7.41 (35.14)	0.90 (5.47)
2018	486	120	366	7.94 (31.87)	10.61 (35.22)	7.06 (30.70)	2.03 (13.35)	6.47 (25.60)	0.57 (3.81)
2019	543	125	418	8.35 (35.32)	8.35 (34.21)	8.35 (35.69)	1.69 (12.95)	3.85 (21.23)	1.04 (9.07)
2020	606	124	482	6.24 (25.86)	6.58 (21.11)	6.15 (26.97)	1.23 (10.25)	2.35 (13.72)	0.94 (9.14)
2021	674	137	537	4.75 (20.95)	6.45 (20.89)	4.32 (20.96)	0.75 (6.10)	1.74 (8.43)	0.50 (5.33)
2022	751	149	602	5.82 (27.42)	4.00 (17.71)	6.27 (29.32)	1.14 (14.39)	1.05 (5.42)	1.16 (15.85)
2023	809	166	643	6.00 (26.56)	5.72 (20.87)	6.07 (27.86)	1.60 (14.36)	2.86 (13.56)	1.28 (14.55)
2024	864	181	683	5.54 (21.89)	5.23 (17.39)	5.62 (22.95)	1.47 (9.82)	2.55 (10.20)	1.18 (9.70)

### Treatments (by age group, 2015–2024)

**Fig. 5:**
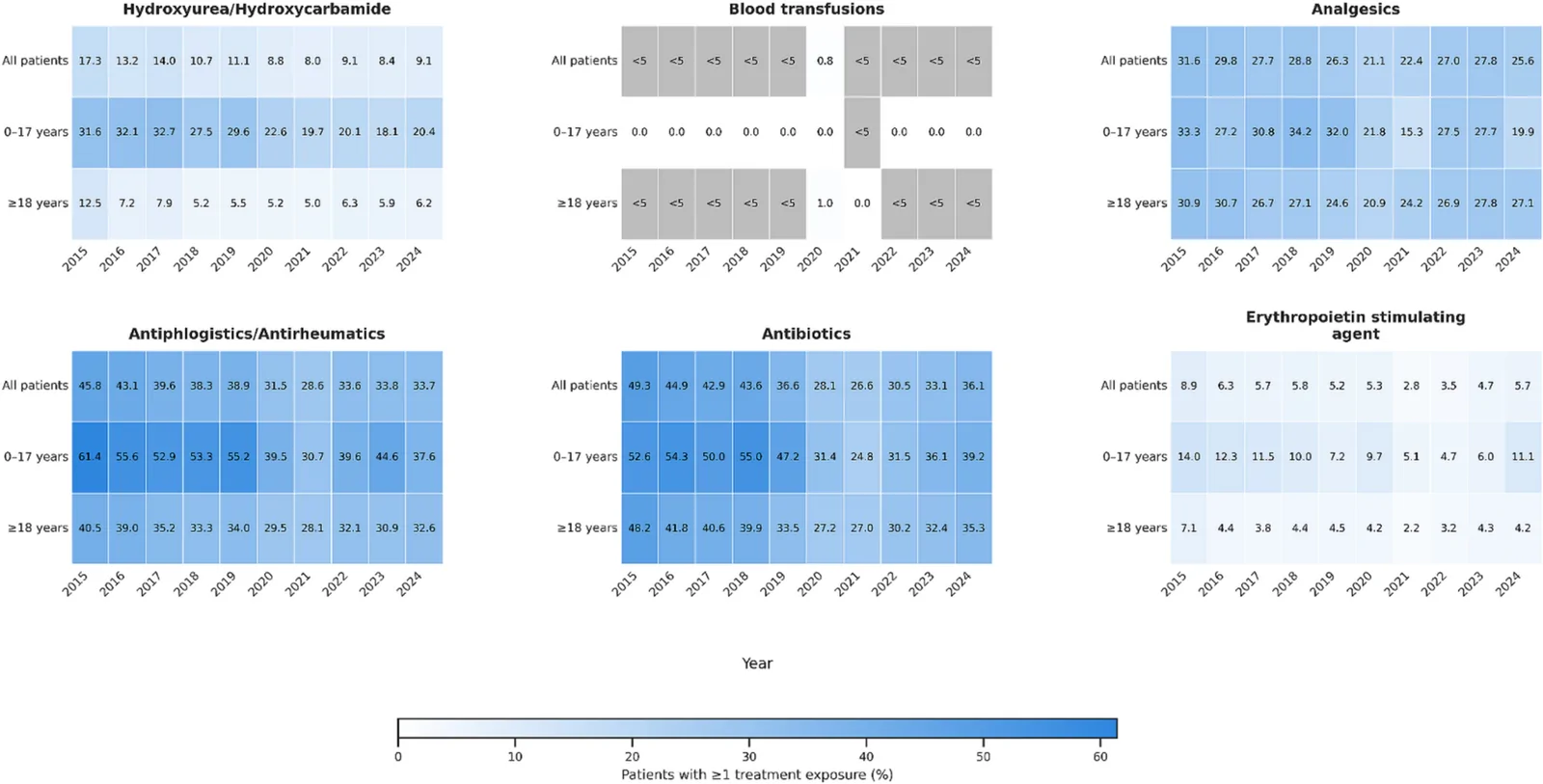
Annual key treatments in patients with SCD (proportion of patients with ≥ 1 documented treatment exposure) in Germany, 2015–2024, by age group. Grey cells indicate cell counts < 5; white cells indicate zero observations. Antibiotics comprise systemic antibacterials (ATC J01). Antiphlogistics/antirheumatics include non‑steroidal anti‑inflammatory drugs (ATC M01). Analgesics include non‑opioid and opioid analgesics (ATC N02 and M01AB)

Annual key treatment exposures are shown in Fig. 5; full treatment results are provided in Online Resource 3. Across years, antibiotics and anti-inflammatory treatments remained common. In all patients, antibiotic exposure decreased from 49.3% in 2015 to 36.1% in 2024; children/adolescents consistently had higher exposure than adults. Antiphlogistics/antirheumatics decreased from 45.8% in 2015 to 33.7% in 2024 and were also more frequently documented in children/adolescents.

Analgesic exposure was common in both age groups and showed a divergent age pattern: in 2024, analgesics were documented more frequently in adults than in children/adolescents (27.1% vs. 19.9%), while levels were similar in 2015 (Fig. 5).

HU/HC exposure declined over time and remained substantially higher in children/adolescents than in adults. In all patients, HU/HC exposure was 17.3% in 2015 and 9.1% in 2024; corresponding values were 31.6% and 20.4% in children/adolescents and 12.5% and 6.2% in adults (Fig. 5).

Erythropoietin stimulating agent exposure was documented throughout the period and was more frequently recorded in children/adolescents than in adults in 2024 (11.1% vs. 4.3%). Blood transfusions were infrequently documented in the year-by-year analyses; where not displayed numerically, values were subject to small-cell handling per data-provider rules (Fig. 5).

## Discussion

This ten-year claims analysis provides population-based evidence on documented SCD epidemiology and routine care patterns in Germany between 2015 and 2024. As expected with a carryforward cohort approach, administrative prevalence increased over time.Among young children, diagnoses were made earlier after 2021 and significant age-related differences were observed in terms of acute complications, co-morbid mental health conditions, the use of inpatient healthcare services, and treatment activities.

### Administrative prevalence (2015–2024)

Administrative national prevalence estimates increased substantially over the observation period, from 2.75 per 100,000 in 2015 to 10.50 per 100,000 in 2024, corresponding to an increase from 225 to 864 prevalent SCD patients in the InGef database. These trends should be interpreted as changes in documented disease burden in healthcare settings rather than biological disease frequency. The carry‑forward prevalence definition is appropriate for a lifelong inherited disorder and reduces undercounting when individuals have intermittent SCD‑coded contacts, but it also implies that temporal changes are seen at least partly as a result of our study design. Our prevalence estimates in each individual year attempt to provide as estimate of the full SCD burden in the German population in that calendar year, including patients who were diagnosed in earlier years and still alive in the current year. The extent to which prevalence increased over calendar years due to a true increase in the general population, due to our carry-forward approach, or due to other factors such as changes in coding practices or healthcare seeking behaviors, cannot be disentangled.

The similarity between primary extrapolations and hospital-corrected sensitivity estimates supports the robustness of our prevalence estimates in each individual calendar year. Furthermore, in the broader German context, the observed increase is consistent with previous German estimates, including at least 2,000 individuals in 2017 reported from registry data and 3,160 individuals in 2019 in a prior claims-based analysis [4, 5]. Although these estimates are not directly comparable because of differences in insured populations and case ascertainment, they support the interpretation that SCD has become increasingly visible in German routine data.

In 2024, although the age-group specific administrative prevalence rate per 100,000 population was higher among children and adolescents, the absolute number of SCD cases was larger among adults (683 versus 181 cases). Possible explanations for the higher prevalence rate among children include age-related differences in the use of medical services and in documentation practices for diagnoses, and/or possible biases in our study sample, including under-representation of older individuals. The newborn screening programme introduced in 2021 [7], may also have contributed to the detection of cases in younger birth cohorts, but cannot explain the age pattern observed prior to 2021.

### Administrative incidence and time to recorded diagnosis in children aged < 6 years

Administrative incidence in children aged < 6 years reflects first recorded SCD diagnoses in claims rather than true incidence at birth. There was some variation in estimated incidence rates over the study period, including a sharp rise in the years 2022 and 2023. This may be associated with the introduction of nationwide newborn screening for SCD in Germany 2021 [12], though results should be interpreted with caution due to the small raw numbers of incident cases identified. A central finding is the reduction in time to recorded diagnosis in young children from 31 months in 2015 to 11 months in 2021–2024. This temporal shift also aligns with the introduction of nationwide newborn screening and is consistent with earlier identification and documentation in routine care [7].

### Burden of disease across the life course: outcomes and comorbidity patterns

Across the study period, infections remained the most frequently documented outcome. Importantly, age‑dependent burden was a consistent observation: children/adolescents more often exhibited patterns consistent with acute, SCD‑related events (including the VOC proxy), whereas adults showed higher documentation of mental health comorbidity and chronic end-organ damage such as retinopathy. This reflects the described natural disease course from predominantly acute complications in childhood and adolescence to a more complex adult disease profile, including progressive end-organ damage and higher documented mental health comorbidity.

Interpretation of VOC findings warrants caution because VOCs are not directly coded in claims data and were approximated using a hospitalization‑based proxy algorithm. Consequently, temporal patterns may reflect not only clinical burden but also care pathways, documentation practices, proxy performance characteristics, and age‑dependent care‑seeking. In adults, some pain episodes may be managed outside hospital settings, potentially lowering hospital‑based proxy capture relative to children/adolescents.

Mental health comorbidity (depression and anxiety) was documented throughout the study period and was consistently more frequently recorded in adults than in children/adolescents. While claims data capture recorded diagnoses rather than symptom burden and no direct general-population comparator was included in this study, the persistent adult predominance supports the relevance of integrating structured psychosocial assessment and support into SCD care pathways [2, 13].

### Healthcare resource utilization: age gradients and changing proportions over time

HCRU patterns complement the outcome findings and further emphasize the life‑course perspective. All‑cause outpatient GP contact remained common over time, while SCD‑related outpatient GP contacts declined in their annual proportion. Hospital‑based measures also showed declining annual proportions in several strata, while age gradients were most pronounced for SCD‑related hospital contacts, which remained clearly higher in children/adolescents than in adults.

Several annual proportions for outcomes, HCRU, and treatments decreased over time while administrative prevalence increased. Because these measures were calculated as annual per-patient proportions, this pattern does not necessarily imply a contradiction. Possible explanations include changes in disease awareness and management, broader capture of patients with milder disease or fewer SCD-related contacts, and changes in documentation or care pathways over time. The descriptive design cannot disentangle these mechanisms, but they should be considered when interpreting temporal trends in routine claims data.

Because LoS summaries are intrinsically influenced by the high share of patients without inpatient stays, interpretation should remain anchored to the broader utilization patterns and the descriptive nature of the analyses.

### Treatments: age‑patterned exposure signatures and cautious cross‑study interpretation

Erythropoietin stimulating agent exposure was observed throughout the study period. In German guidelines, erythropoietin is described mainly in selected clinical contexts, such as renal anemia/chronic kidney disease and certain transfusion-related hemolytic complications; accordingly, ESA signals in routine claims data likely reflect heterogeneous indications that cannot be differentiated further in claims data [14, 15].

Cross-study comparison of HU/HC estimates should be made cautiously. In Germany-specific routine data, a prior AOK claims analysis reported increasing hydroxyurea/hydroxycarbamide use, reaching approximately 44% of patients in 2019 [5]. Divergent estimates between that study and the present analysis are most likely explained by differences in insured populations and case ascertainment rather than by treatment capture itself.

The observed age-patterns also support the notion that a life-course approach to SCD care, which takes account of different clinical manifestations at different life stages, is important. For example, pediatric/adolescent care was characterized by higher acute SCD-related contact burden, whereas adult care showed higher documentation of mental health comorbidity and distinct treatment exposure patterns, highlighting the importance of continuity and coordinated care across life stages. Transition from pediatric to adult services is clinically important, but transition-specific effects were not analyzed separately in this study.

### Strengths and limitations

Strengths of this study include the use of a large statutory health insurance claims dataset with multi‑year coverage, enabling consistent assessment of documented epidemiology and routine care patterns across calendar years, and the use of a hospital discharge–based correction as a robustness check for national extrapolation.

Limitations include our carry-forward cohort approach for the calculation of prevalence, meaning that estimates for individual calendar years attempt to represent SCD prevalence in the general population as completely as possible, but that temporal trends over time should be interpreted with caution (i.e. are partly due to our study design). Other limitations include those inherent to claims data analyses, e.g. that claims data are collected for reimbursement and are susceptible to misclassification due to coding variability. The comparatively frequent use of ICD-10-GM code D57.8 should be interpreted cautiously, as it does not allow genotype-level interpretation and may reflect non-specific coding. Key clinical information (e.g., genotype, severity, laboratory parameters) is not available, limiting clinical phenotyping. VOCs required a proxy definition, making results sensitive to documentation and care pathway changes. Estimates for early-life incidence and time to recorded diagnosis were based on children aged < 6 years irrespective of age at entry into insurance coverage. Although this approach improved sample size and interpretability compared with a more restrictive full-coverage cohort, it may have introduced selection bias and limits direct interpretation as birth incidence.

### Implications and conclusions

In summary, this claims‑based analysis indicates increasing documented SCD prevalence in Germany, earlier recorded diagnosis timing in young children in the post‑2021 period, and a substantial burden of disease reflected in complications, mental health comorbidity and SCD‑related healthcare utilization. These findings support life‑course–oriented care pathways that integrate pediatric acute care needs, structured psychosocial assessment and support in adulthood, and continuity planning across the transition from pediatric to adult services. At the same time, the coexistence of rising administrative prevalence with lower annual proportions for some outcomes, HCRU measures, and treatments indicates that claims-based time trends reflect changes in recorded burden and care patterns, not necessarily changes in disease severity alone. Complementary data sources, such as registries or clinically enriched datasets, are therefore important to validate and extend claims-based evidence on SCD epidemiology and burden in Germany.

## Supplementary Information

Below is the link to the electronic supplementary material.

Supplementary Material 1

Supplementary Material 2

Supplementary Material 3

Supplementary Material 4

Supplementary Material 5

## Data Availability

The data analyzed in this study were obtained from the InGef research database. In accordance with German laws, data protection requirements, and contractual restrictions, the underlying individual-level claims data are not publicly available.
