# Supplementary material for: Sickle cell disease in Germany – trends in epidemiology, healthcare utilization and disease burden from 2015 to 2024: a claims data analysis
Source: Ann Hematol. 2026 Oct 23;105(11):478. doi: 10.1007/s00277-026-07308-2 (PMC13690866; doi:10.1007/s00277-026-07308-2)
Supplement: Supplementary file 1 [file 277_2026_7308_MOESM1_ESM.pdf]

Supplementary Information

Article title
Sickle cell disease in Germany – trends in epidemiology, healthcare utilization and disease burden from 2015 to 2024: a claims data analysis

Journal
Annals of Hematology

Authors
Nils Schulze; Moritz Lehne; Anja Cengia; Klara Sonnie Hinrichsmeyer; Agnes Kisser; Ferras Alashkar; Caoimhe Cawley

Ferras Alashkar and Caoimhe Cawley share last authorship.

Corresponding author
Nils Schulze
Medical University Brandenburg Theodor Fontane
Institute of Health Services and Health Systems Research
Immanuel Clinic Rüdersdorf
Seebad 82/83
15562 Rüdersdorf bei Berlin
Germany

E-mail: nils.schulze@mhb-fontane.de

Online Resource 1

Protocol codes and operational definitions for clinical outcomes, VOCs, HCRU, and treatments

This online resource provides the detailed code lists and operational definitions referenced in the Methods section of the manuscript. Codes are based on the German claims-data setting and include ICD-10-GM, OPS, ATC, PZN, and EBM/GOP sources where applicable.

Table OR1-1. Overview of study variables

Category	Variables / operational description
Baseline characteristics	• Demographics: sex, age, health insurance status<br>• SCD case definition: at least one primary inpatient diagnosis and/or at least one confirmed outpatient SCD diagnosis (ICD-10-GM D57.0, D57.1, D57.2, D57.8) documented by a hematologist (German specialty code 27, 35, or 41), or at least two confirmed outpatient SCD diagnoses documented by two independent physicians during the study period 01/01/2015 to 31/12/2024

Category	Variables / operational description
Clinical outcomes	• Vaso-occlusive crises (VOCs)<br>• Pneumonia / acute chest syndrome (ACS)<br>• Sepsis<br>• Splenic sequestration<br>• Splenectomy<br>• Cholecystectomy<br>• Acute anemia<br>• Organ failure: heart failure, renal failure, respiratory failure, hepatic failure<br>• Stroke<br>• Retinopathy<br>• Osteonecrosis / osteoporosis<br>• Mental health comorbidity: depression, anxiety<br>• Thromboembolism<br>• Pulmonary hypertension<br>• Priapism<br>• Infections<br>• Joint replacement<br>• Mortality
Healthcare resource utilization (HCRU)	• Outpatient GP visits (all-cause and SCD-related)<br>• Outpatient specialist visits (all-cause and SCD-related)<br>• Hospitalizations: ambulatory hospitalizations (all-cause and SCD-related), inpatient hospitalizations (all-cause and SCD-related), length of stay
Treatments	• Disease-modifying treatment: hydroxyurea / hydroxycarbamide, crizanlizumab, voxelotor, allogeneic stem-cell transplantation (SCT)<br>• Infection prevention / treatment: antibiotics (including penicillin, amoxicillin, ceftriaxone, azithromycin, cefotaxime)<br>• Iron chelation therapy: deferoxamine, deferiprone, deferasirox<br>• Supportive and symptomatic treatments: anti-inflammatory / antirheumatic agents (Ibuprofen), coxibs (Cele-, Rofe-, Valde-, Pare-, Etori-, Lumira-, Polmacoxib), analgesics (Paracetamol, Diclofenac, Metamizole, Opioids (Tramadol, Tilidine, Morphinum hydrochloricum, Fenanyl, Hydromoprhone, Pethidine)); erythropoiesis-stimulating agents<br>• Blood transfusions<br>• Vitamin D

Table OR1-2. Clinical outcomes and operational definitions

Outcome	Code(s) / operational definition
Vaso-occlusive crises (VOC proxy)	Any hospital stay (irrespective of stay duration) with one of the following primary or secondary diagnoses: SCD with crisis (ICD-10-GM D57.0), pneumonia / acute chest syndrome (ICD-10-GM J12-J18), or priapism (ICD-10-GM N48.3).
Pneumonia / acute chest syndrome (ACS)	ICD-10-GM J12-J18
Sepsis	ICD-10-GM A02.1, A20.7, A22.7, A39.2-A39.4, A40, A41, A42.7, B00.7
Splenic sequestration	ICD-10-GM D73

Outcome	Code(s) / operational definition
Splenectomy	OPS 5-413
Cholecystectomy	OPS 5-511
Acute anemia	ICD-10-GM D62
Heart failure	ICD-10-GM I50, I01.8, I09.8, I02.0, I97.1
Acute kidney failure	ICD-10-GM N17
Respiratory failure	ICD-10-GM J96
Liver failure	ICD-10-GM K72
Stroke	ICD-10-GM I60-I64, G45.8, G45.9
Retinopathy	ICD-10-GM H35
Osteonecrosis / osteoporosis	ICD-10-GM M87, M90, M81
Depression	ICD-10-GM F32, F33, F34
Anxiety	ICD-10-GM F40, F41
Thromboembolism	ICD-10-GM I80, I82
Pulmonary hypertension	ICD-10-GM I27
Priapism	ICD-10-GM N48.3
Infection	ICD-10-GM A00-B99
Joint replacement	OPS 5-82
Mortality	Based on date of death recorded in claims data

Note: ICD codes include relevant subordinate subcodes where applicable.

Table OR1-3. HCRU variables and operational definitions

Variable	Definition
All-cause outpatient GP visits	All outpatient visits (any ICD-10-GM code) documented by a physician with specialty code 01, 02, 03 (adults) or 34 (children).
SCD-related outpatient GP visits	All outpatient visits documented by a physician with specialty code 01, 02, 03 (adults) or 34 (children) and an SCD diagnosis (ICD-10-GM D57.0, D57.1, D57.2, D57.8) in the outpatient case associated with the visit.
All-cause outpatient specialist visits	All outpatient visits (any ICD-10-GM code) documented by a hematology-related physician specialty code: 27 (adults) or 35 / 41 (children).
SCD-related outpatient specialist visits	All outpatient visits documented by a hematology-related physician specialty code: 27 (adults) or 35 / 41 (children) and an SCD diagnosis (ICD-10-GM D57.0, D57.1, D57.2, D57.8) in the outpatient case associated with the visit.
All-cause ambulatory hospitalizations (≤1 day)	Hospital stays with any ICD-10-GM code and a length of stay of ≤1 day.
SCD-related ambulatory hospitalizations (≤1 day)	Hospital stays with an SCD diagnosis (ICD-10-GM D57.0, D57.1, D57.2, D57.8) and a length of stay of ≤1 day.
All-cause inpatient hospitalizations (>1 day)	Hospital stays with any ICD-10-GM code and a length of stay of >1 day.
SCD-related inpatient hospitalizations (>1 day)	Hospital stays with an SCD diagnosis (ICD-10-GM D57.0, D57.1, D57.2, D57.8) and a length of stay of >1 day.
Length of stay	Summarized as mean (SD) for all-cause and SCD-related inpatient stays.

Note: A patient may contribute more than one contact or hospitalization within a given study period.

Table OR1-5. Treatment categories and corresponding ATC / OPS / EBM codes

Treatment category / treatment	ATC code	OPS / EBM code
**Disease-modifying treatment**		
Hydroxyurea / hydroxycarbamide	L01XX05	
Voxelotor	B06AX03	
Crizanlizumab	B06AX01	

Treatment category / treatment	ATC code	OPS / EBM code
Allogeneic stem-cell transplantation (general)		8-863
Allogeneic, non-HLA-identical related donor		8-805.2, 5-411.2
Allogeneic, non-HLA-identical unrelated donor		8-805.3, 5-411.3
Allogeneic, HLA-identical related donor		8-805.4, 5-411.4
Allogeneic, HLA-identical unrelated donor		8-805.5, 5-411.5
**Infection prevention / treatment**		
Antibiotics	J01	
Penicillin	J01CE02	
Amoxicillin	J01CA04	
Ceftriaxone	J01DD04	
Azithromycin	J01FA10	
Cefotaxime	J01DD01	
**Iron chelation therapy**		
Deferoxamine	V03AC01	
Deferiprone	V03AC02	
Deferasirox	V03AC03	
**Supportive and symptomatic treatment**		
Anti-inflammatory / antirheumatic agents	M01	
Ibuprofen	M01AE01	
Coxibs	M01AH	
Celecoxib	M01AH01	
Rofecoxib	M01AH02	
Valdecoxib	M01AH03	
Parecoxib	M01AH04	
Etoricoxib	M01AH05	
Lumiracoxib	M01AH06	
Polmacoxib	M01AH07	
Analgesics	N02	
Paracetamol	N02BE01	
Diclofenac	M01AB05	
Metamizole	N02BB02	
Opioids	N02A	
Tramadol	N02AX02	
Tilidine	N02AX01	
Morphine hydrochloride	N02AA01	
Fentanyl	N02AB03	
Hydromorphone	N02AA03	
Pethidine	N02AB02	
Erythropoiesis-stimulating agents	B03XA01	
Blood transfusions		OPS 8-800, 8-801; EBM 02110, 02111

Treatment category / treatment	ATC code	OPS / EBM code
Vitamin D	A11CC	
