# Supplementary material for: Sickle cell disease in Germany – trends in epidemiology, healthcare utilization and disease burden from 2015 to 2024: a claims data analysis
Source: Ann Hematol. 2026 Oct 23;105(11):478. doi: 10.1007/s00277-026-07308-2 (PMC13690866; doi:10.1007/s00277-026-07308-2)
Supplement: Supplementary file 2 [file 277_2026_7308_MOESM4_ESM.pdf]

Supplementary Information

Article title
Sickle cell disease in Germany – trends in epidemiology, healthcare utilization and disease burden from 2015 to 2024: a claims data analysis

Journal
Annals of Hematology

Authors
Nils Schulze; Moritz Lehne; Anja Cengia; Klara Sonnie Hinrichsmeyer; Agnes Kisser; Ferras Alashkar; Caoimhe Cawley

Ferras Alashkar and Caoimhe Cawley share last authorship.

Corresponding author
Nils Schulze
Medical University Brandenburg Theodor Fontane
Institute of Health Services and Health Systems Research
Immanuel Clinic Rüdersdorf
Seebad 82/83
15562 Rüdersdorf bei Berlin
Germany

E-mail: nils.schulze@mhb-fontane.de

Online Resource 4 – STROBE

STROBE Statement—Checklist of items that should be included in reports of ***cohort studies***

	Item No	Recommendation
**Title and abstract**	1	(a) Title; Abstract (Methods)
		(b) Abstract (Background, Methods, Results, Conclusion)
**Introduction**		
Background/rationale	2	Introduction, paras. 1–4
Objectives	3	Introduction, final paragraph
**Methods**		
Study design	4	Methods – Study design and data source
Setting	5	Methods – Study design and data source
Participants	6	(a) Methods – Study population and SCD case definition; Results – Study population; Table 1
		(b) Not applicable (no matched design)
Variables	7	Methods – Study population and SCD case definition; Epidemiological measures; Clinical outcomes and HCRU; Online Resource 1
Data sources/ measurement	8*	Methods – Study design and data source; Clinical outcomes and HCRU; Online Resource 1
Bias	9	Methods – National extrapolation and hospital-correction sensitivity analysis; Discussion – Strengths and limitations
Study size	10	Methods – Study design and data source; Results – Study population
Quantitative variables	11	Methods – Administrative prevalence; Administrative incidence; Time to recorded diagnosis; Stratification, summary measures, and small-cell suppression
Statistical methods	12	(a) Methods – National extrapolation and hospital-correction sensitivity analysis; Stratification, summary measures, and small-cell suppression

		(b) Methods – Stratification, summary measures, and small-cell suppression; Results – stratified analyses by age group
		(c) Not explicitly reported
		(d) Not applicable (routine claims data; no conventional loss to follow-up analysis)
		(e) Methods – Time to recorded diagnosis (<6 years); National extrapolation and hospital-correction sensitivity analysis; Results – incidence paragraph; Discussion – Strengths and limitations
**Results**		
Participants	13*	(a) Results – Study population; Table 1
		(b) Not applicable / not separately reported for administrative database study
		(c) Table 1
Descriptive data	14*	(a) Results – Study population; Table 2
		(b) Not explicitly reported
		(c) Not explicitly reported
Outcome data	15*	Results – Administrative prevalence; Administrative incidence; Clinical outcomes; HCRU; Treatments; Tables 1–3; Figs. 1–5; Online Resource 3
Main results	16	(a) Results – Administrative prevalence and incidence; Table 1; Figs. 1–2 (descriptive estimates with 95% CIs; no confounder-adjusted analyses)
		(b) Methods – Stratification, summary measures, and small-cell suppression
		(c) Not applicable
Other analyses	17	Results – age-group analyses; hospital-corrected estimates; supplementary full-coverage cohort; Online Resources 2–3
**Discussion**		
Key results	18	Discussion, first paragraph
Limitations	19	Discussion – Strengths and limitations
Interpretation	20	Discussion; Implications and conclusions
Generalisability	21	Discussion – Administrative prevalence; Strengths and limitations; Implications and conclusions
**Other information**		
Funding	22	Statements and Declarations – Funding

*Give information separately for exposed and unexposed groups.

Note: An Explanation and Elaboration article discusses each checklist item and gives methodological background and published examples of transparent reporting. The STROBE checklist is best used in conjunction with this article (freely available on the Web sites of PLoS Medicine at http://www.plosmedicine.org/, Annals of Internal Medicine at http://www.annals.org/, and Epidemiology at http://www.epidem.com/). Information on the STROBE Initiative is available at http://www.strobe-statement.org.
