# Supplementary material for: Sickle cell disease in Germany – trends in epidemiology, healthcare utilization and disease burden from 2015 to 2024: a claims data analysis
Source: Ann Hematol. 2026 Oct 23;105(11):478. doi: 10.1007/s00277-026-07308-2 (PMC13690866; doi:10.1007/s00277-026-07308-2)
Supplement: Supplementary file 3 [file 277_2026_7308_MOESM5_ESM.pdf]

Supplementary Information

Article title
Sickle cell disease in Germany – trends in epidemiology, healthcare utilization and disease burden from 2015 to 2024: a claims data analysis

Journal
Annals of Hematology

Authors
Nils Schulze; Moritz Lehne; Anja Cengia; Klara Sonnie Hinrichsmeyer; Agnes Kisser; Ferras Alashkar; Caoimhe Cawley

Ferras Alashkar and Caoimhe Cawley share last authorship.

Corresponding author
Nils Schulze
Medical University Brandenburg Theodor Fontane
Institute of Health Services and Health Systems Research
Immanuel Clinic Rüdersdorf
Seebad 82/83
15562 Rüdersdorf bei Berlin
Germany

Online Resource 5 – RECORD checklists

The RECORD statement – checklist of items, extended from the STROBE statement, that should be reported in observational studies using routinely collected health data.

	**Item No.**	**STROBE items**	**Location in manuscript where items are reported**	**RECORD items**	**Location in manuscript where items are reported**
**Title and abstract**					
	1	(a) Indicate the study's design with a commonly used term in the title or the abstract (b) Provide in the abstract an informative and balanced summary of what was done and what was found	(a) Title; Abstract (Methods)<br>(b) Abstract (Background, Methods, Results, Conclusion)	RECORD 1.1: The type of data used should be specified in the title or abstract. When possible, the name of the databases used should be included.<br><br>RECORD 1.2: If applicable, the geographic region and timeframe within which the study took	RECORD 1.1: Title; Abstract (Methods)<br>RECORD 1.2: Title; Abstract (Background, Methods)<br>RECORD 1.3: Not applicable (no database linkage performed)

				place should be reported in the title or abstract.<br><br>RECORD 1.3: If linkage between databases was conducted for the study, this should be clearly stated in the title or abstract.	
**Introduction**					
Background rationale	2	Explain the scientific background and rationale for the investigation being reported	Introduction, paras. 1–4		
Objectives	3	State specific objectives, including any prespecified hypotheses	Introduction, final paragraph		
**Methods**					
Study Design	4	Present key elements of study design early in the paper	Methods – Study design and data source		
Setting	5	Describe the setting, locations, and relevant dates, including periods of recruitment, exposure, follow-up, and data collection	Methods – Study design and data source		
Participants	6	*(a) Cohort study* - Give the eligibility criteria, and the sources and methods of selection of participants. Describe methods of follow-up<br>*Case-control study* - Give the eligibility criteria, and the sources and methods of case ascertainment and control selection. Give the rationale for the choice of cases and controls<br>*Cross-sectional study* - Give the eligibility criteria, and the sources and methods of selection of participants<br><br>*(b) Cohort study* - For matched	(a) Methods – Study population and SCD case definition; Results – Study population; Table 1<br>(b) Not applicable (no matched design)	RECORD 6.1: The methods of study population selection (such as codes or algorithms used to identify subjects) should be listed in detail. If this is not possible, an explanation should be provided.<br><br>RECORD 6.2: Any validation studies of the codes or algorithms used to select the population should be referenced. If validation was conducted for this study and not published elsewhere, detailed methods and results should be provided.<br><br>RECORD 6.3: If the study involved linkage of databases, consider use of a	RECORD 6.1: Methods – Study population and SCD case definition; Clinical outcomes (VOC proxy); Online Resource 1<br>RECORD 6.2: Not explicitly reported<br>RECORD 6.3: Not applicable (no database linkage performed)

		studies, give matching criteria and number of exposed and unexposed<br>*Case-control study* - For matched studies, give matching criteria and the number of controls per case		flow diagram or other graphical display to demonstrate the data linkage process, including the number of individuals with linked data at each stage.	
Variables	7	Clearly define all outcomes, exposures, predictors, potential confounders, and effect modifiers. Give diagnostic criteria, if applicable.	Methods – Study population and SCD case definition; Epidemiological measures; Clinical outcomes and HCRU; Online Resource 1	RECORD 7.1: A complete list of codes and algorithms used to classify exposures, outcomes, confounders, and effect modifiers should be provided. If these cannot be reported, an explanation should be provided.	Online Resource 1; Methods – Study population and SCD case definition; Clinical outcomes
Data sources/ measurement	8	For each variable of interest, give sources of data and details of methods of assessment (measurement).<br>Describe comparability of assessment methods if there is more than one group	Methods – Study design and data source; Clinical outcomes and HCRU; Online Resource 1		
Bias	9	Describe any efforts to address potential sources of bias			Methods – National extrapolation and hospital-correction sensitivity analysis; Discussion – Strengths and limitations
Study size	10	Explain how the study size was arrived at			Methods – Study design and data source; Results – Study population
Quantitative variables	11	Explain how quantitative variables were handled in the analyses. If applicable, describe which groupings were chosen, and why			Methods – Administrative prevalence; Administrative incidence; Time to recorded diagnosis; Stratification, summary measures, and small-cell suppression

Statistical methods	12	(a) Describe all statistical methods, including those used to control for confounding<br>(b) Describe any methods used to examine subgroups and interactions<br>(c) Explain how missing data were addressed<br>(d) *Cohort study* - If applicable, explain how loss to follow-up was addressed<br>*Case-control study* - If applicable, explain how matching of cases and controls was addressed<br>*Cross-sectional study* - If applicable, describe analytical methods taking account of sampling strategy<br>(e) Describe any sensitivity analyses			(a) Methods – National extrapolation and hospital-correction sensitivity analysis; Stratification, summary measures, and small-cell suppression<br>(b) Methods – Stratification, summary measures, and small-cell suppression; Results – stratified analyses by age group<br>(c) Not explicitly reported<br>(d) Not applicable (routine claims data; no conventional loss to follow-up analysis)<br>(e) Methods – Time to recorded diagnosis (<6 years); National extrapolation and hospital-correction sensitivity analysis; Results – incidence paragraph; Discussion – Strengths and limitations
Data access and cleaning methods		..		RECORD 12.1: Authors should describe the extent to which the investigators had access to the database population used to create the study population.	RECORD 12.1: Not explicitly reported
				RECORD 12.2: Authors should provide information on the data cleaning methods used in the study.	RECORD 12.2: Not explicitly reported
Linkage		..		RECORD 12.3: State whether the study included person-level,	RECORD 12.3: Not applicable (no

				institutional-level, or other data linkage across two or more databases. The methods of linkage and methods of linkage quality evaluation should be provided.	database linkage performed)
**Results**					
Participants	13	(a) Report the numbers of individuals at each stage of the study (*e.g.*, numbers potentially eligible, examined for eligibility, confirmed eligible, included in the study, completing follow-up, and analysed)<br>(b) Give reasons for non-participation at each stage.<br>(c) Consider use of a flow diagram	(a) Results – Study population; Table 1<br>(b) Not applicable / not separately reported for administrative database study<br>(c) Table 1	RECORD 13.1: Describe in detail the selection of the persons included in the study (*i.e.*, study population selection) including filtering based on data quality, data availability and linkage. The selection of included persons can be described in the text and/or by means of the study flow diagram.	Methods – Study population and SCD case definition; Results – Study population; Table 1
Descriptive data	14	(a) Give characteristics of study participants (*e.g.*, demographic, clinical, social) and information on exposures and potential confounders<br>(b) Indicate the number of participants with missing data for each variable of interest<br>(c) *Cohort study* - summarise follow-up time (*e.g.*, average and total amount)	(a) Results – Study population; Table 2<br>(b) Not explicitly reported<br>(c) Not explicitly reported		
Outcome data	15	*Cohort study* - Report numbers of outcome events or summary measures over time<br>*Case-control study* - Report numbers in each exposure	Results – Administrative prevalence; Administrative incidence; Clinical outcomes; HCRU; Treatments; Tables 1–3; Figs. 1–5; Online Resource 3		

		category, or summary measures of exposure<br>*Cross-sectional study* - Report numbers of outcome events or			

		summary measures			
Main results	16	(a) Give unadjusted estimates and, if applicable, confounder-adjusted estimates and their precision (e.g., 95% confidence interval). Make clear which confounders were adjusted for and why they were included<br>(b) Report category boundaries when continuous variables were categorized<br>(c) If relevant, consider translating estimates of relative risk into absolute risk for a meaningful time period	(a) Results – Administrative prevalence and incidence; Table 1; Figs. 1–2 (descriptive estimates with 95% CIs; no confounder-adjusted analyses)<br>(b) Methods – Stratification, summary measures, and small-cell suppression<br>(c) Not applicable		
Other analyses	17	Report other analyses done—e.g., analyses of subgroups and interactions, and sensitivity analyses	Results – age-group analyses; hospital-corrected estimates; supplementary full-coverage cohort; Online Resources 2–3		
**Discussion**					
Key results	18	Summarise key results with reference to study objectives	Discussion, first paragraph		
Limitations	19	Discuss limitations of the study, taking into account sources of potential bias or imprecision. Discuss both direction and magnitude of any potential bias	Discussion – Strengths and limitations	RECORD 19.1: Discuss the implications of using data that were not created or collected to answer the specific research question(s). Include discussion of misclassification bias, unmeasured confounding, missing data, and changing eligibility over time, as they pertain to the study being reported.	Discussion – Strengths and limitations
Interpretation	20	Give a cautious overall interpretation of results considering objectives, limitations, multiplicity of analyses, results from similar studies, and other relevant evidence	Discussion; Implications and conclusions		

Generalisability	21	Discuss the generalisability (external validity) of the study results		Discussion – Administrative prevalence; Strengths and limitations; Implications and conclusions
**Other Information**				
Funding	22	Give the source of funding and the role of the funders for the present study and, if applicable, for the original study on which the present article is based		Statements and Declarations – Funding
Accessibility of protocol, raw data, and programming code		..	RECORD 22.1: Authors should provide information on how to access any supplemental information such as the study protocol, raw data, or programming code.	Statements and Declarations – Data availability; Supplementary information

*Reference: Benchimol EI, Smeeth L, Guttmann A, Harron K, Moher D, Petersen I, Sørensen HT, von Elm E, Langan SM, the RECORD Working Committee. The REporting of studies Conducted using Observational Routinely-collected health Data (RECORD) Statement. *PLoS Medicine* 2015; in press.

*Checklist is protected under Creative Commons Attribution (CC BY) license.
